# The Current Role of Lymph Node Dissection in the Management of Renal Cell Carcinoma

**DOI:** 10.1155/2011/816926

**Published:** 2011-06-07

**Authors:** Joseph Edmund Jamal, Thomas William Jarrett

**Affiliations:** Department of Urology, The George Washington University Medical Faculty Associates, 2150 Pennsylvania Avenue, NW, Washington, DC 20037, USA

## Abstract

The role of lymph node dissection remains controversial in the surgical management of renal cell carcinoma. Incidental renal masses are being diagnosed at increasing rates due to the routine use of CT scans. Despite the increase in incidental diagnosis of renal masses, 20% to 30% of patients present with metastatic disease. Currently, surgeons do not routinely perform lymph node dissection unless there is gross evidence of lymphadenopathy, as patients without clinical evidence of lymphadenopathy rarely have positive nodes at the time of surgery. Patients with metastatic disease to the regional lymph nodes have a poor overall prognosis. However, some evidence supports a therapeutic benefit of lymphadenectomy in these patients. Further, the staging information gained from diagnosing lymph node involvement may allow for the use of new agents to treat metastatic disease and effect outcomes.

## 1. Introduction

The role of lymph node dissection (LND) remains controversial in the surgical management of renal cell carcinoma (RCC) [1–3]. To date, there is no definitive data which indicates an overall survival advantage imparted by performing LND in patients with RCC. Additionally, a complete LND during radical nephrectomy (RN) adds significant time, potential morbidity, and requires dissection of and around the great vessels. What is clear is that in patients with renal cell carcinoma without evidence of distant metastases, the presence of lymph node-only metastases is associated with a poor prognosis [4–10]. For this reason, identifying patients at risk for positive lymph nodes at the time of surgical treatment of their renal mass remains essential. 

Recently, the first prospective, randomized trial was conducted by the European Organization for Research and Treatment of Cancer (EORTC) to compare the long-term results of radical nephrectomy alone (*n* = 389) versus radical nephrectomy with complete lymphadenectomy (*n* = 383) for patients with clinically N0, M0 disease [1]. They concluded that LND is not therapeutic in the routine management of RCC, but nor did it increase the morbidity of surgical management. This has spurred significant debate in the urologic oncology community [3, 11, 12]. Indeed, this study was aimed at determining the long-term outcomes in patients with clinically localized disease, and found that 70% of patients had pT1 or pT2 disease. The majority of these patients were therefore unlikely to benefit from LND in the first place. Therefore, it is important to emphasize that although being a landmark study, it does not negate the role for LND in RCC. With the recent availability of new adjuvant agents, prospective adjuvant protocol studies nearing completion, and neoadjuvant protocols currently under study, the importance of LND may change, either as a therapeutic or staging procedure. The topic of LND therefore requires further discourse.

In clinical practice, the role of LND in the management of RCC remains controversial due to variability of lymphatic drainage of the kidney, the absence of lymphatic involvement in many patients with disseminated disease, and the lack of definitive evidence of any survival benefit imparted by LND [1]. We address the current controversies regarding the role of lymphadenectomy in the surgical management of renal tumors in this review. Ultimately, the therapeutic benefit of LND in RCC, whether it be beneficial by itself or in that it may be diagnostic of metastatic disease resulting in initiation of adjuvant chemotherapy, may only be realized in a select subset of patients.

## 2. Epidemiology and Relevance

Renal cell carcinoma comprises 2% to 3% of malignant neoplasms in adults. In the United States, 31,000 new cases of RCC are diagnosed annually, and 11,900 patients die of disease [13, 14]. There is a male-to-female ratio of 3 : 2 [13], and a 10% to 20% higher incidence in African Americans [15]. The majority of cases of RCC are believed to be sporadic, with only 4% familial according to National Cancer Institute estimates. Approximately 20% to 30% of patients with RCC present with metastatic disease [16], but ranges from 3% in surgical series to 63.6% in autopsy series [16]. Of these patients with metastatic disease, historically, 40% have distant metastases only without evidence of lymph node involvement, 50% have both distant metastases and lymph node involvement, and approximately 3–10% present with lymph node involvement only [1, 17–19]. Additionally, one-third of patients with localized RCC will eventually develop recurrence or progression. There has been downward stage migration, as well as increasing incidence of RCC due to extensive utilization of cross-sectional imaging. 

In combination, the clinical stage and pathologic grade of the tumor is highly predictive of positive lymph node metastases. When LND is performed, a number of studies have shown that positive lymph nodes have an independent adverse effect on outcome, irrespective of other variables [20–22]. Patients with node-positive disease have 5-year survival rates ranging from 5% to 35% [23]. 

## 3. Templates for Lymph Node Dissection

Renal lymphatic drainage is variable, adding to the controversy of performing LND at the time of RN. There is currently no consensus on the anatomic extent of LND. This makes it exceedingly difficult to compare studies as there is a clear lack of standardization. Further, many studies do not delineate the template used for LND. A “standard” LND for RCC on the left side includes the paraaortic and preaortic nodes from the crus of the diaphragm to the inferior mesenteric artery (IMA). On the right, a “standard” LND includes the paracaval and precaval nodes from the adrenal vein along the vena cava, also down to the level of the IMA. An “extended” LND adds the interaortocaval nodes down to the bifurcation of the great vessels on both sides, with the inclusion of retrocaval nodes for right-sided primary tumors. 

Crispen and colleagues recently examined their series of patients with LND and evaluated the lymphatic drainage patterns for recommendations for surgical templates [24]. Of 169 patients who fit the criteria for LND, 64 (38%) were found to have metastatic disease to their retroperitoneal lymph nodes, with the median of 6 lymph nodes removed with LND. For right-sided tumors, the primary lymphatic drainage was the paracaval, precaval, retrocaval, and interaortocaval lymph nodes. For left-sided tumors, the primary lymphatic drainage was the paraaortic, preaortic, retroaortic, and interaortocaval lymph nodes. They advocate for a standard removal of all the lymphatic tissue in these primary lymphatic landing zones. When examining their data of patients with positive nodal disease, left-sided tumors corresponded with 76% of left hilar and 62% of paraaortic positive lymph nodes (of patients with positive nodal disease). On the right side, patients with positive lymph node involvement were found to occur in 57% of paracaval and 43% of right hilar nodes. On both sides, lymph nodes from the renal hilum were not always involved in patients with nodal disease, supporting the argument against merely sampling the renal hilar lymph nodes as being sufficient. Based on these findings, they recommend that when lymphadenectomy is performed in patients without palpable disease, that left-sided disease LND includes paraaortic and interaortocaval lymph nodes, and right-sided disease LND includes paracaval and interaortocaval lymph nodes. 

Morbidity of LND has largely been found to be minimal when compared to nephrectomy alone. Retrospective review has shown little difference in morbidity of LND [25]. Additionally, the recent prospective EORTC 30881 trial also showed no appreciable difference in morbidity between their two randomized groups. They did not comment on the additional length of time the LND added, nor the total number of lymph nodes removed. They included morbidities such as bleeding >1 liter, pleural injury, infection, bowel injury, embolism, and lymph fluid drainage, and found no significant differences between patients who had LND and those who did not. Siminovitch and colleagues [26] performed a direct comparison of extended, hilar, and regional LND templates. They found no differences in morbidity or survival rates between the three different LND templates. Regardless, LND remains a complex procedure, and carries risk for serious intraoperative complications. Great care must be taken when performing the procedure to ensure minimal morbidity.

## 4. Indications and Assessment

Indications for LND at the time of RN are not generally clear. Signs of lymphadenopathy or evidence of locally advanced disease on cross-sectional imaging, warrants LND. Palpable lymphadenopathy, or evidence of bulky lymph nodes with laparoscopy, at the time of RN can be indications for LND. Unfortunately, radiographic lymphadenopathy only modestly correlates with metastatic involvement, with 32%–43% of nodes >1 cm harboring cancer [27, 28]. Other studies have shown that 16%–42% of lymph nodes suspicious on are falsely positive [1, 18, 29]. Many of these nodes are inflammatory in nature, and therefore no benefit is imparted by their removal. The difficulties lie in determining the lymphatic drainage patterns of each kidney, which lymph nodes are potentially positive, and to what extent a lymphadenectomy should be performed. 

Nomograms have been developed to aid in risk stratification. One nomogram had 78% accuracy by incorporating patient age, radiographic tumor size, and symptoms [28]. Another preoperative nomogram, which included 4844 patients, found a concordance index of 0.76 when including symptoms at presentation, radiographic lymphadenopathy, tumor size, and hematuria [30]. Neither of these nomograms have gained widespread acceptance.

Crispen and colleagues evaluated the performance of LND in high-risk clear cell RCC patients between 2002 and 2006 [24]. They had previously identified five intraoperative pathological features which were considered high-risk for nodal metastases, and performed LND if at least two of these risk factors were present intraoperatively. These were nuclear grade 3 or 4, sarcomatoid component, tumor size ≥10 cm, tumor stage pT3 or pT4, and coagulative tumor necrosis. Of 169 RNs that had 2 high-risk intraoperative factors, 63 (38%) had nodal metastases. 

Others have novel approaches to expanding indications for LND. Bex and colleagues evaluated the role of sentinel node detection in patients with RCC [31]. Previous series indicate that 58–95% of patients with lymph node disease have associated hematogenous spread [32, 33], prompting Bex and colleagues to assess the feasibility of identifying a sentinel node to aid in staging. Eight patients with cT1 cN0 cM0 RCC had their tumors injected (percutaneously under ultrasound guidance) with a radionuclide tracer, ^99m^Tc-nanocolloid. Both lymphoscintigraphy and SPECT/CT were performed to determine the anatomic location of the sentinel node, as there is variable lymphatic drainage of the kidney. Surgery was then performed the following day utilizing intraoperative gamma probes to identify the radioactive nodes. Of the eight patient tumors injected, six patients were found to have identifiable sentinel nodes on scintigraphy. In two patients, no identifiable drainage of the radiotracer was found. In this small study, an identifiable sentinel node could be found in 75% of tumors. The authors suggest that this may be helpful in patients in which biopsy of the sentinel node could clarify the extent of lymphatic involvement, which could have diagnostic and therapeutic implications [31]. 

Ming and colleagues evaluated the utility in performing frozen section analysis of enlarged lymph nodes during RN for RCC [34]. They performed frozen section analysis on lymph nodes >1 cm, before undertaking an extended lymphadenectomy. Of 702 consecutive patients, 114 had evidence of enlarged lymph nodes or palpably enlarged nodes and underwent frozen section analysis. On final pathology, they found that 78 patients (68.4%) with enlarged lymph nodes did not harbor cancer while 36 (31.6%) did have nodal metastases. Of these 36 patients with nodal disease on final pathology, 32 had positive findings on frozen section, resulting in positive predictive value of 100% and negative predictive value of 95%. The study concludes that it would be reasonable to avoid LND in patients with clinically localized RCC in whom frozen section analysis of enlarged lymph nodes reveals no evidence of malignant disease. However, this does not indicate any therapeutic advantage to the procedure in patients with lymph node disease. 

## 5. Outcomes of LND

Patients with clinically localized RCC have not been shown to benefit from routine LND [1]. The EORTC 30881 trial confirmed that after appropriate clinical staging, in patients with clinical N0M0 disease, the incidence of unsuspected lymph-node metastases was only 4.0%. When compared with nephrectomy alone, they showed no advantage to performing LND on patients with clinically localized disease with regards to overall survival, local regional progression, or distant progression. Of the 346 patients in whom LND was performed, 51 had palpably enlarged lymph nodes at the time of surgery. Of these, only 10 patients (20%) had lymph node metastases. Remarkably, of the 311 remaining patients without palpable nodes, only 4 patients (1%) were shown to have metastatic disease to their lymph nodes (*P* < .001). The potential benefits of staging for these patients are also minimal. These statements are especially true for more low-risk disease (T1-2, N0, M0). 

The more difficult patient population to address is those with clinically localized, high-risk disease (T3-4, N0, M0). The root of this difficulty stems from the fact that there is substantial risk of hematogenous dissemination of disease, and relatively low risk of node-only involvement. The therapeutic benefit of LND in these patients is questionable at best. As previously discussed, Blute and colleagues identified 5 risk factors (including clinical stage T3-4) for lymph node metastases. The presence of at least 2 risk factors was associated with a 15-fold higher incidence of regional lymph node involvement. Although difficult to implement, this is reasonable approach for these patients in whom occult disease may be cured. 

Patients who present with clinical nodal disease should have LND performed. It is relatively infrequent for patients to present with isolated positive nodes, without distant metastases, but is estimated to occur in 3% to 10% of cases [1, 17–19]. It is essential, therefore, to accurately rule out distant metastatic disease if one suspects lymph node involvement only. Survival of these patients improves when LND is performed compared to nephrectomy alone [32]. Further, the overall survival of these patients who undergo LND with radical nephrectomy is far superior to patients who present with distant metastases, and in fact more closely approximates the survival of patients with T3, N0, M0 disease [35, 36]. Giuliani and colleagues showed that 5-year survival in patients with lymph node only disease was 47.9%, compared with 7% for patients with distant metastases. An extended lymph node dissection, as described previously, is recommended in these patients.

Patients with metastatic disease may benefit only slightly, if at all, from LND at the time of nephrectomy. Nodal metastases are poorly responsive to immunotherapy, so removal of grossly positive nodes is reasonable. Although extended lymphadenectomy may theoretically be beneficial, there is no evidence to support this, and lymphadenectomy must be balanced with the patient's comorbidities and performance status. A useful, evidence-based algorithm was offered by Godoy and colleagues [16] that can be utilized in the decision to perform LND at the time of radical nephrectomy. With the advent of new tyrosine kinase inhibitors and cytokines, the value of LND in patients with metastatic disease may increase in the near future, either as adjuvant or neoadjuvant therapy. 

## 6. Current State of Targeted Therapies

Previously, systemic treatment of patients with metastatic RCC was limited to cytokine therapy with interleukin- (IL-) 2 or interferon- (IFN-) *α*, because of mRCC's general resistance to chemotherapy. High-dose IL-2 remains the only treatment to produce durable remissions, and it should be considered in healthy, appropriately chosen candidates as adjuvant therapy. New targeted therapies have been developed through genetic studies of familial von Hippel Lindau disease. Specifically, molecular cell signaling pathways involving the vascular endothelial growth factor (VEGF) and the mammalian target of rapamycin (mTOR) pathways have been targeted with tyrosine kinase inhibitors (TKIs) and mTOR inhibitors, respectively. 

Node-only metastatic RCC is rare, and no specific data of these patients treated with targeted therapies currently exists. Instead, we suggest treating these patients similar to those having a solitary metastasis. Based on current data, sunitinib (TKI) is justified as a first-line standard of care for patients with clear cell RCC [37, 38]. Another first-line therapy for clear cell RCC is combination therapy with bevacizumab plus interferon-*α* based on recent phase III data. Patients with nonclear cell metastatic RCC should be enrolled in clinical trials; however, they have been treated with TKIs, mTOR inhibitors, and even chemotherapy with gemcitabine and cisplatin for metastatic sarcomatoid RCC. 

Currently, randomized controlled studies have been initiated to determine the current role and optimal timing of removal of the primary tumor in patients with primary metastatic disease, since historically cytoreductive nephrectomy followed by cytokine treatment had better overall survival than cytokine therapy alone. Therefore, participation in a clinical trial is currently considered as the best management of metastatic RCC patients presenting with a resectable primary and synchronous metastases. In patients with more advanced disease, anecdotal evidence has suggested that neoadjuvant targeted therapies disease can improve feasibility of cytoreductive nephrectomy, reduce nodal involvement, and facilitate lymphadenectomy by improving the planes of dissection [39]. We look forward to the further development of a targeted therapy regimen for patients with metastatic disease. 

## 7. Conclusion

Lymphadenectomy at the time of radical nephrectomy is rarely performed and is not supported in the majority of patients with renal tumors, especially with the downward stage migration of disease. It does not appear to confer a survival advantage in patients who have clinically localized disease. However, LND at the time of nephrectomy is not associated with significantly increased morbidity [1] and is warranted if there is sufficient clinical suspicion on staging CT, or if bulky lymphadenopathy is found at the time of surgery. Although lymphadenectomy undoubtedly improves the accuracy of staging and provides better prognostic information, there is little impact on progression-free or overall survival in patients with clinically localized disease. Risk factors may increase the likelihood of lymph node metastases, and may be a way to better determine patients at risk for nodal involvement. Future studies with novel targeted therapies may increase the indications for LND further. 
